# MRI Assessment of Motor Capabilities in Patients with Duchenne Muscular Dystrophy According to the Motor Function Measure Scale

**DOI:** 10.3390/tomography8020076

**Published:** 2022-04-01

**Authors:** Vasily Suslov, Galina Suslova, Sergey Lytaev

**Affiliations:** 1 Department of Rehabilitation, Saint Petersburg State Pediatric Medical University, 194100 Saint Petersburg, Russia; ga.suslova@gpmu.org; 2 Department of Normal Physiology, Saint Petersburg State Pediatric Medical University, 194100 Saint Petersburg, Russia; mail@physiolog.spb.ru

**Keywords:** Duchenne muscular dystrophy, Motor Function Measure, MRI, echo time, repetition time, weighted images

## Abstract

The research was aimed on the study of motor capabilities on the Motor Function Measure (MFM) scale in ambulant and non-ambulant patients with Duchenne muscular dystrophy, and to conduct a correlation analysis between the results of the MFM scale and Magnetic Resonance Imaging (MRI) data. A total of 46 boys who had genetically confirmed Duchenne muscular dystrophy (age from 2.1 to 16.7 years) and were in clinical rehabilitation were investigated. An assessment was performed according to the Motor Function Measure scale (subsections D1, D2, D3, and the total score), an MRI obtaining T1-VI of the muscles of the pelvic girdle was conducted, and the thighs and lower legs were further assessed in terms of the severity of fibrous-fat degeneration according to the Mercuri scale. In ambulant patients, the ability to stand up and move (D1) was 74.4%, axial and proximal motor functions (D2)—97.6%, distal motor functions (D3)—96.2%, and total score was 87.9%. In non-ambulant patients, the ability to stand up and move (D1) was 1.7%, axial and proximal motor functions (D2)—47%, distal motor functions (D3)—67.5%, and the total score—33.1%. A high inverse correlation (r = −0.7, *p* < 0.05) of the MRI data of the pelvic girdle and thighs with tasks D1, as well as a noticeable inverse correlation with tasks D2 (r = −0.6, *p* < 0.05) of the scale MFM, were revealed in the ambulant group of patients. In the non-ambulant group of patients, the MRI data of the lower legs muscles were characterized by a high inverse correlation (r = −0.7, *p* < 0.05) with tasks D3 and a noticeable inverse correlation (r = −0.6, *p* < 0.05) with tasks D1 of the MFM scale. Conclusion: The Motor Function Measure scale allows effective assessment of the motor capabilities of patients with Duchenne muscular dystrophy at different stages of the disease, which is confirmed by visualization of fibro-fatty muscle replacement.

## 1. Introduction

Duchenne muscular dystrophy (DMD) is the most severe and common form among childhood muscular dystrophies due to a mutation in the dystrophin gene (DMD) [1,2]. The incidence is 3.3 per 10 thousand newborn boys [3,4,5]. The disease is characterized by a steady progressive course, severe weakness, and atrophy with a predominant lesion of the skeletal muscles of the pelvic girdle, thighs, and legs, which leads to early disability and loss of ability to move independently at the age of about 12 years [6,7]. As the disease progresses, the involvement of the axial and proximal muscles of the upper limbs and the development of cardiological, respiratory, and orthopedic complications are characteristic [8,9,10,11]. The foregoing factors necessitate the use of reliable, effective, and affordable clinical and instrumental methods for assessing the condition of skeletal muscles, applicable at all stages of the course of DMD [12,13,14,15,16,17]. The use of scales for determining the motor abilities of patients allows you to effectively evaluate these indicators, not only at the moment, but also in dynamics [18,19,20]. The Motor Function Measure (MFM) scale allows you to assess the percentage of motor capabilities in patients with neuromuscular diseases, and is divided into three groups of tasks aimed at assessing verticalization and walking (task D1), and axial, proximal (task D2), and distal (task D3) motor functions [21,22,23]. However, the final results may be affected by factors such as age, cognitive abilities and motivation of the subject, as well as the experience and subjective opinion of the researcher themself [24]. This necessitates objective instrumental methods for assessing the state of the muscular system in patients with neuromuscular diseases, which primarily include magnetic resonance imaging (MRI) [25,26,27,28,29].

Currently, in the field of MR imaging of skeletal muscles, various quantitative methods for assessing the fibro-fatty degeneration of skeletal muscles in DMD and other neuromuscular diseases are widely used, such as three-point Dixon, T2 MSME, IDEAL-CPMG, etc., demonstrating high sensitivity in the dynamic monitoring of the disease progression. However, the presence of technical and software limitations in many clinics requires consideration of the possibilities of semi-quantitative methods for determining the severity of fat replacement as an alternative widely available assessment method [25,30,31].

Based on the foregoing, the aim of the study was to assess motor capabilities on the MFM scale in patients with DMD, and to conduct a correlation analysis between the results of the MFM scale and MRI data.

## 2. Materials and Methods

A total of 46 patients (boys) with genetically confirmed Duchenne muscular dystrophy at the age from 2.1 to 16.7 years were examined. All patients were divided into 2 groups, depending on motor capabilities. The first group (with intact walking ability) consisted of 34 patients aged 2.1 to 11.5 years (mean age = 6.7 years). The second group (without the possibility of independent movement) consisted of 7 patients aged 10.2 to 16.7 years (mean age = 13.9 years).

All patients were assessed for motor abilities on the Motor Function Measure (MFM) scale: tasks D1 (lifting and moving), D2 (axial and proximal muscles of the upper and lower extremities), D3 (distal muscles of the upper and lower extremities), and the total score, including all 3 groups of tasks. For subjects over 7 years old, a version of the scale consisting of 32 tasks (MFM-32) was used. For children under 7 years old, an abbreviated scale of 20 tasks was used (MFM-20).

Magnetic resonance imaging of skeletal muscles for 32 patients was performed. The study was carried out on a Philips Ingenia 1.5T magnetic resonance imager using an external body coil with T1-VI (TE = 9 ms, TR = 500 ms, flip angle = 90°, and slice thickness = 10 mm, slice gap = 15 mm). Investigation of the muscles of the pelvic girdle (mm. Gluteus maximus, medius, minimus), hips (m. Adductor longus, m. Adductor brevis, m. Adductor magnus, m. Squadriceps, m. Biceps femoris, m. Semitendinosus, m. Semimembranosus), and lower legs (m. gastrocnemius, m. soleus, m. peroneus, m. tibialis anterior, m. tibialis posterior, m. extensor digitorum longus, m. extensor hallucis longus, m. flexor digitorum longus, m. flexor hallucis longus) with two sides was performed. The data obtained on a semi-quantitative Mercuri scale were evaluated (Table 1) [32]. Subsequently, the total points for the muscles of the pelvic girdle and hips, legs, and the total points of all the studied muscles of the lower extremities were calculated. The general quantitative characteristics of the study are presented in Table 2.

The MFM score was performed by a board-certified neurologist, one of the authors of this article who specializes in neuromuscular diseases and has received specialized training in the use of the MFM score. The same specialist, who has completed training in and has long-term experience of muscle MRI—including international internships and professional specialist certification—performed and assessed the MRI. In order to avoid errors, strict clinical evaluation of the patients was carried out before muscle MRI.

For the sum of points on the Mercuri scale and the MFM scale (tasks D1, D2, D3, and the total score), the average values were calculated and the Spearman correlation analysis was carried out with the assessment of the correlation strength on the Cheddock scale, and the level of statistical significance was calculated.

Statistical analysis using the IBM SPSS 23.0 software (Statistics v.23) was performed. Means and confidence intervals were calculated. Correlation analysis of clinical data and age was carried out according to the Pearson method for the group with the preserved ability to move, and according to the Spearman method for the group without the ability to move. Correlation analysis of semi-quantitative MRI data was carried out according to the Spearman method.

The research was performed in the clinic of St. Petersburg State Pediatric Medical University from January 2017 to October 2020. The research was approved by the decision of the local ethics committee, protocol No. 1/2 of 16 January 2017. 

### Study Limitations

Several limitations in this study were considered. Firstly, among the 46 patients with Duchenne muscular dystrophy, MRI muscle studies were performed only in 32 patients due to early childhood and behavioral disorders in some patients. Secondly, drug sedation was not performed during muscle MRI due to the high risk of rhabdomyolysis in patients with DMD. Thirdly, the study was performed including only the lower extremities in patients with DMD. At the same time, the Motor Function Measure scale allows you to comprehensively evaluate the functions of both the lower and upper limbs, which is of great importance when working with patients in the late stages of the disease. Carrying out a correlation analysis between the functions of the upper extremities and the severity of fatty muscle degeneration (according to MRI data of patients with advanced stages of the disease) will be relevant to future studies.

Quantitative evaluation of FF (%) and water T2, as well as evaluation of the cross-sectional area, were also carried out by us. Nevertheless, according to the authors, due to the frequent technical and software limitations that are encountered in many centers, it is of great importance to evaluate the possibilities of alternative methods for assessing muscle damage, including semi-quantitative methods. MRI and clinical assessment were performed and evaluated by a specialist investigator. In addition, MRI data were evaluated by staff radiologists. In a blind comparison, the differences in scores were practically the same.

The small number of patients is due to the relatively low incidence of DMD (3–5 per 10,000 newborn boys) and a number of serious difficulties and limitations when working with patients at non-outpatient stages of the disease, which leads to a small sample (*n* = 7). For statistical analysis, in this case nonparametric statistical methods were chosen in order to reduce the probability of error. The number of patients able to move makes it possible to obtain reliable information about the severity of the course of the disease.

## 3. Results

### 3.1. Assessment of Motor Capabilities on the MFM Scale

In the group of patients capable of walking, the greatest preservation of the functions of the distal extremities and fine motor skills (D3) was noted, the average values of which were 96.2%. In terms of the functions of the proximal and axial departments (D2), the average values were 97.6%.

**Figure 1:**
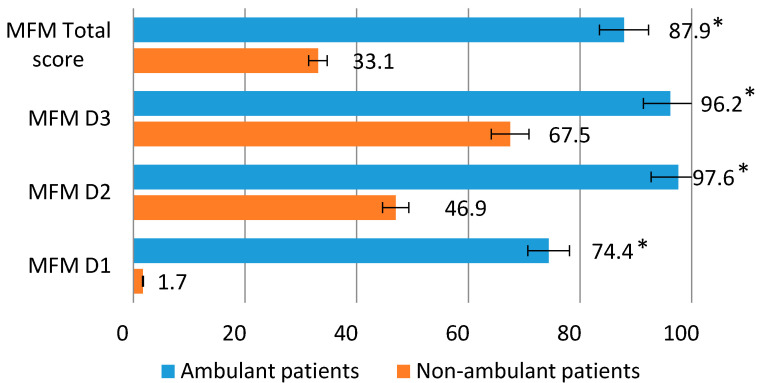
Average values of the Motor Function Measure scale (%) for patients able and unable to walk independently. * *p* ≤ 0.01.

The ability to lift and move (D1) in this group was reduced to 74.4%. The total score of motor capabilities averaged 87.9%. The group of patients incapable of walking was characterized by the highest preservation of the distal extremities (D3) (average values 67.5%) and proximal and axial muscles (D2) (average values 47.0%). The ability to lift and move (D1) averaged only 1.7%. The total score of motor capabilities was on average 33.1% (Figure 1).

**Figure 2:**
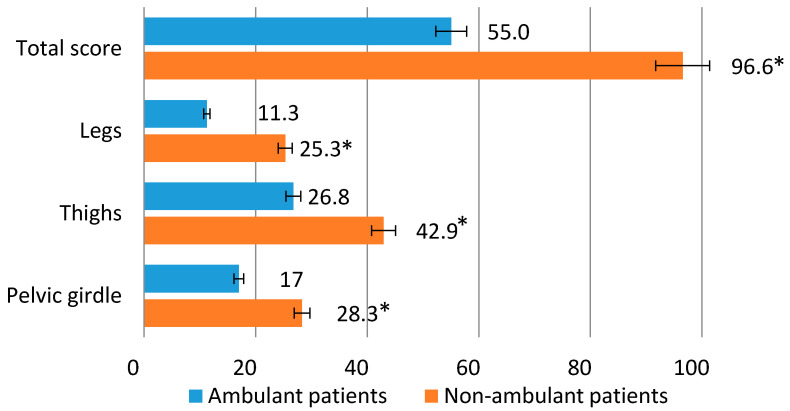
The average Mercuri score for patients with ability and inability to walk independently. * *p* ≤ 0.01.

The average Mercuri score for a group of patients with intact mobility was 17 points for the pelvic muscles, 26.8 points for the muscles of the hips, 11.3 points for the muscles of the legs, and 55.0 points for the total score of the lower extremities (pelvic girdle, hips and legs). For a group of patients unable to move, the average values for the muscles of the pelvic girdle were 28.3 points, for the muscles of the hips 43 points, for the muscles of the legs 25.3 points, and for the total score of the lower extremities 96.6 points (Figure 2).

### 3.2. Correlation of the Motor Function Measure Scale with MRI Data of Skeletal Muscles of the Lower Extremities

When assessing the correlation in the group of patients capable of independent movement, a high inverse correlation was revealed between the D1 tasks of the MFM scale and the results of MRI of the pelvic girdle and hips (r = −0.7, *p* ≤ 0.01), as well as the sum of the MRI scores for the lower extremities (pelvic girdle, hips and lower leg) (r = −0.7, *p* ≤ 0.01). Tasks D1 also showed a noticeable inverse correlation with the MRI data of the calf muscles (r = −0.6, *p* ≤ 0.05). Tasks D2 were characterized by a noticeable inverse correlation with the results of MRI of the muscles of the pelvic girdle and hips (r = −0.5, *p* ≤ 0.05). Assignments D3 also showed a noticeable inverse correlation with MRI of the leg muscles (r = −0.6, *p* ≤ 0.05). When analyzing the total score of the MFM scale and the sum of the scores of the pelvic girdle, hips, and lower legs according to MRI, a noticeable inverse correlation was revealed (r = −0.6, *p* ≤ 0.05) (Table 3).

The group of patients unable to move independently was characterized by a high inverse correlation only when conducting a correlation analysis between tasks D3 and the results of MRI of the skeletal muscles of the legs (r = −0.7, *p* ≤ 0.05). Tasks D1 showed a noticeable inverse correlation with MRI of the leg muscles (r = −0.6, *p* ≤ 0.05). No correlation was found between tasks D1 and the results of MRI of the muscles of the pelvic girdle and hips, as well as with the total score for the lower extremities (r = −0.3). Tasks D2 and the results of MRI of the muscles of the pelvic girdle and hips were characterized by moderate inverse correlation (r = −0.4). The total MFM score and the sum of the MRI scores (pelvic girdle, hips and lower legs) were characterized by a noticeable inverse correlation (r = −0.5, *p* ≤ 0.05) (Table 4).

During the correlation analysis, a high inverse correlation of the age of patients able to move with the subsections D1, D2, and the general score of the MFM scale (r = −0.7, *p* ≤ 0.01), as well as a noticeable inverse correlation with the subsection D3 (r = −0.5, *p* ≤ 0.05), were revealed. The age of patients incapable of independent movement was characterized by a high inverse correlation with all subsections and the total score of the MFM scale (r = −0.7–0.9, *p* ≤ 0.01).

During the correlation analysis, the age of patients who are able to move independently was characterized by a strong positive correlation with the severity of fibro-fatty degeneration of the muscles of the pelvic girdle and hips and a total score on the Mercuri scale (r = 0.7, *p* ≤ 0.01), and a noticeable positive correlation with damage to the muscles of the legs (r = 0.5, *p* ≤ 0.05). The age of patients unable to move was characterized by a high direct correlation with all MRI data (r = 0.7–0.9, *p* ≤ 0.01) (Table 5).

**Figure 3:**
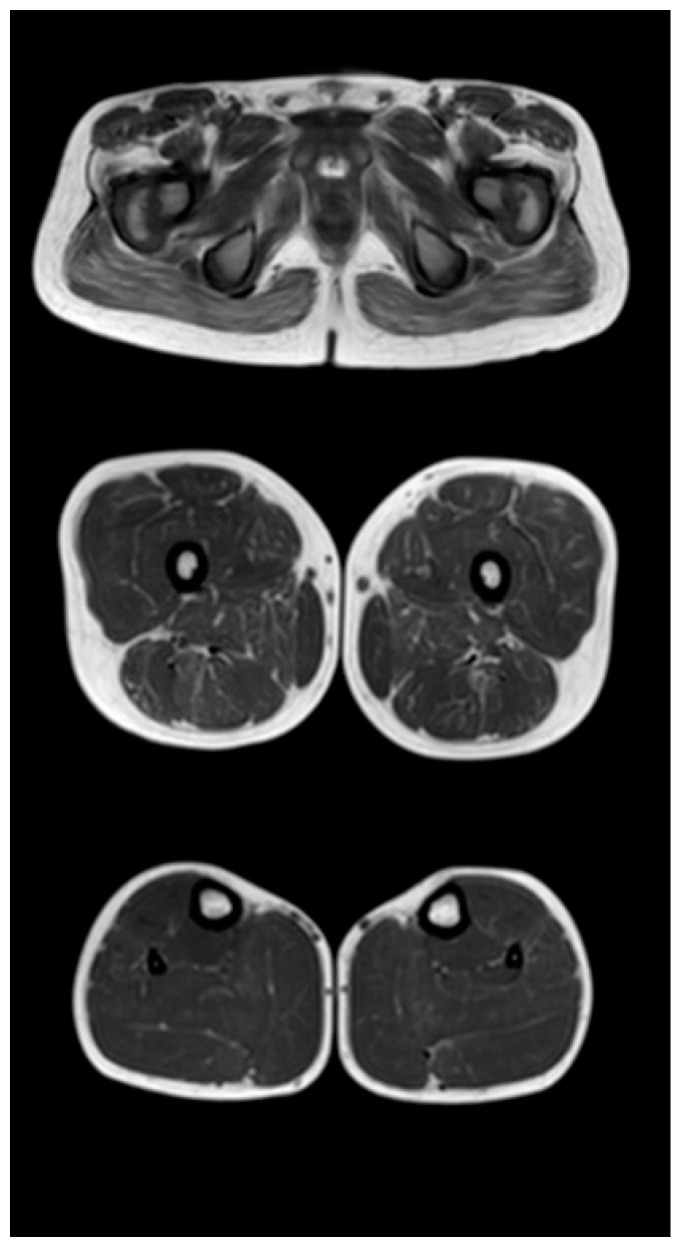
Pattern of involvement of the muscles of the lower extremities in a patient at an early outpatient stage of the disease (6.1 years). From top to bottom: the pelvic level, the femoral level, and the level of the legs.

**Figure 4:**
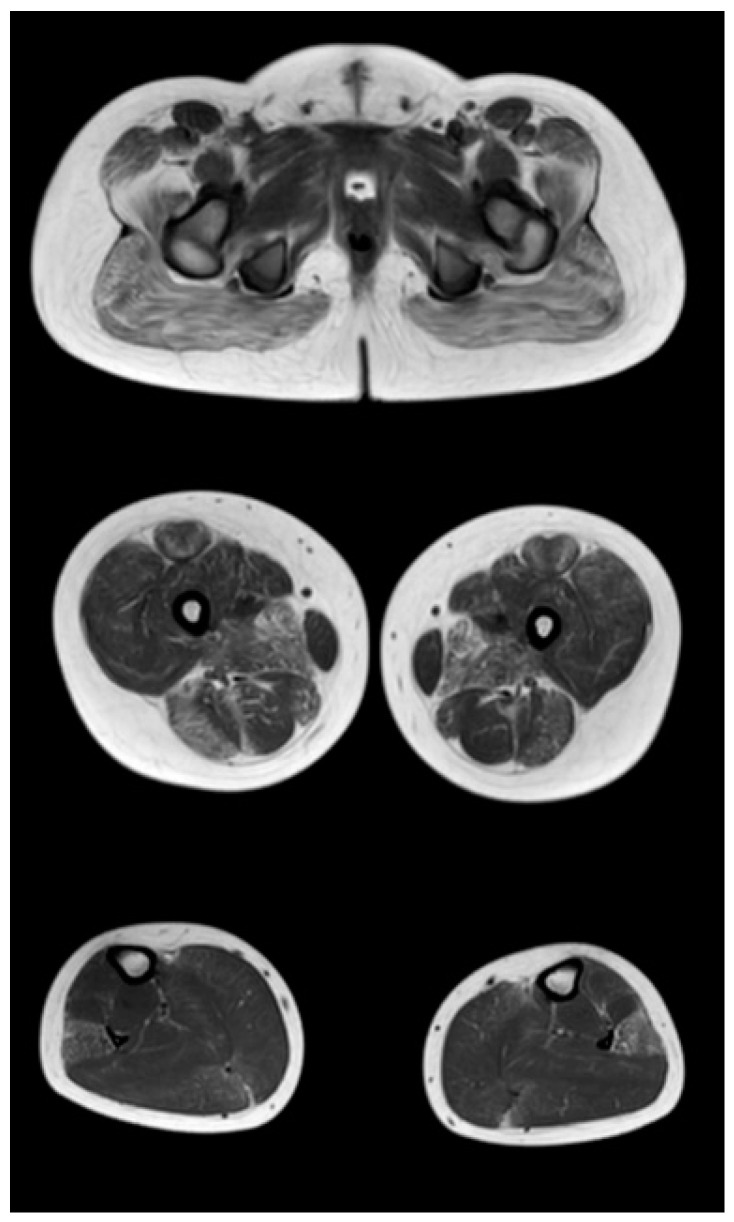
Pattern of involvement of the muscles of the lower extremities in a patient at the late outpatient stage of the disease (9.4 years). From top to bottom: the pelvic level, the femoral level, and the level of the legs.

**Figure 5:**
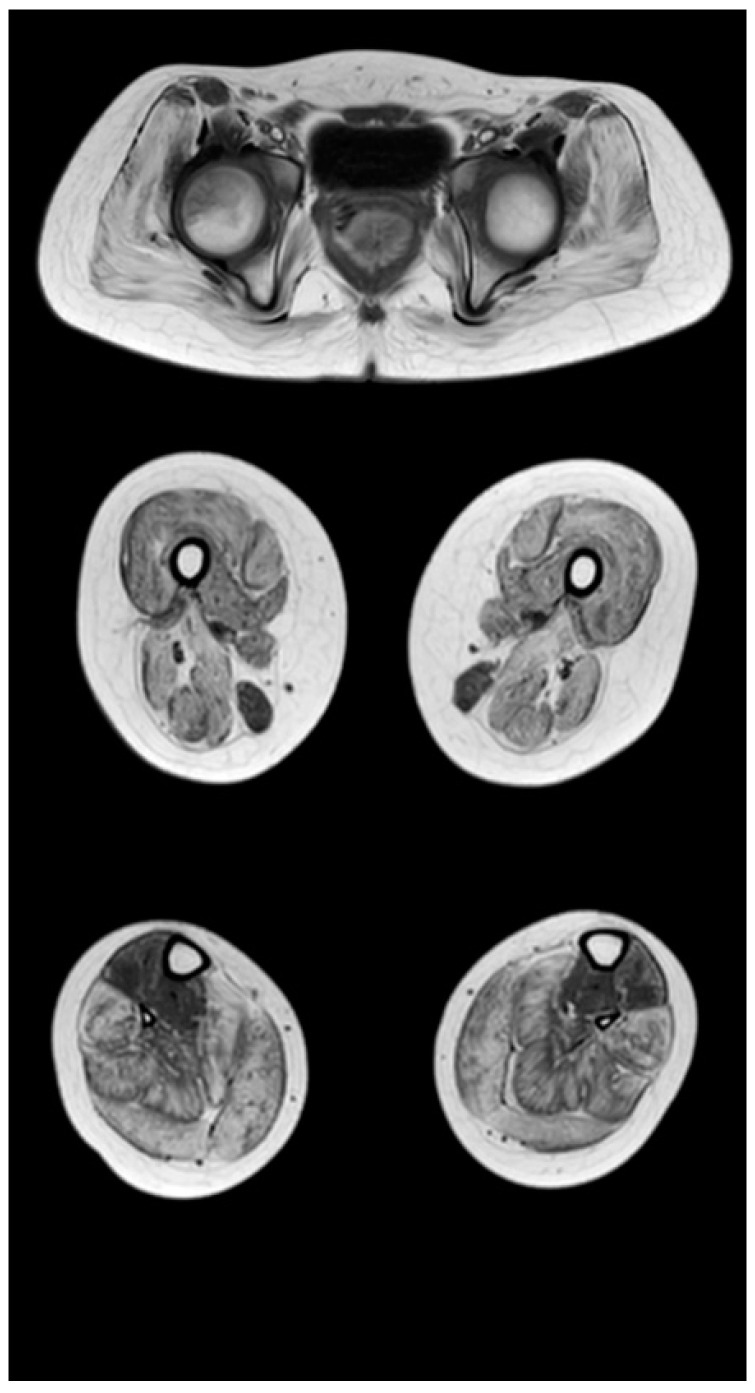
Pattern of involvement of the muscles of the lower extremities in a patient at a late non-ambulatory stage of the disease (13 years). From top to bottom: the pelvic level, the femoral level, and the level of the legs.

Figure 3, Figure 4 and Figure 5 show the main changes in the muscles at different stages of the disease. The muscles of the pelvic girdle are characterized by early symmetric fibro-fatty degeneration of the gluteus maximus muscles, in the Figure characterized by stage 2b on the Mercuri scale. In the hips, the early lesion pattern is characterized by primary involvement of the adductor mausors (stage 2a). It is also characterized by slight diffuse changes in the quadriceps muscles and the posterior group of muscles of the thighs. In the muscles of the legs at the early outpatient stage, nonspecific diffuse muscle fat substitution is characteristic of stage 1 (Figure 3).

At the late outpatient stage, the muscles of the pelvic girdle are characterized by heavy fat substitution of the gluteus maximus and middle muscles (stages 3 and 2b), as well as the muscles straining the fascia lata of the thigh (stage 2b), while the long and short adductor muscles are preserved (Figure 4). In the thighs, progressive fibro-fatty degeneration of the adductor muscles (stage 3), the biceps femoris (stage 2b on the right and stage 2a on the left), semitendinosus, semimembranosus, and quadriceps femoris muscle (stage 2a) is visualized. Thin and sartorius muscles at this stage are characterized by minimal changes or complete preservation (0–1 stages). At the late outpatient stage, the legs are characterized by a pronounced progressive lesion of the long peroneal muscles (stage 3) and of the medial and lateral heads of the gastrocnemius muscles (stage 2a).

Figure 5 shows the pattern of lower limb muscle involvement in a patient in the late non-ambulatory stage of the disease. The late non-ambulatory stage is characterized by total fibro-fatty degeneration of the muscles of the pelvic girdle, hips, and legs (stage 4). Noteworthy is the negative pattern—complete preservation of the gracilis muscle (stage 0) and the anterior muscle group of the legs—of the anterior and posterior tibial muscles (stages 2a and 1, respectively).

**Figure 6:**
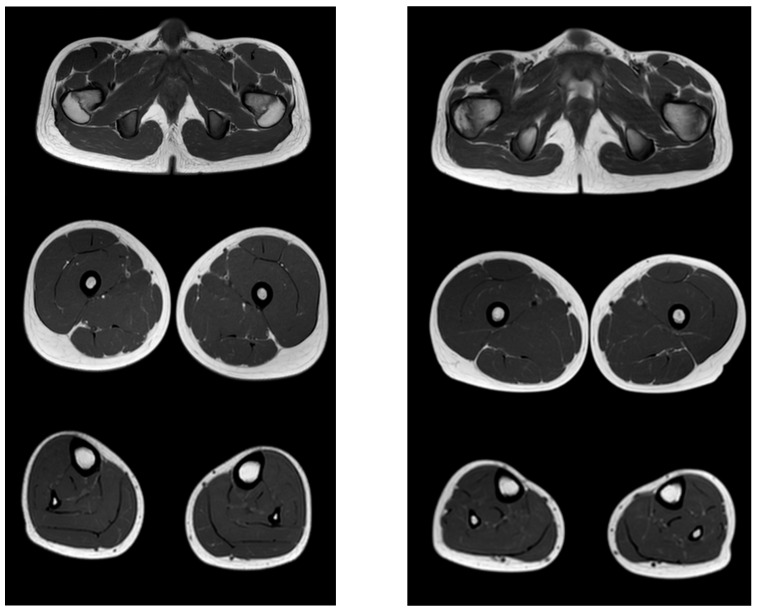
MR images of healthy volunteers 13 years old (**left**) and 8.3 years old (**right**). From top to bottom: the pelvic level, the femoral level and the level of the legs.

Comparative results of assessing the severity of fibro-fatty degeneration of muscles according to the Mercuri scale (point) of patients in accordance with clinical examples (Figure 3, Figure 4 and Figure 5) are presented in Table 6. For comparison of MR patterns in DMD, Figure 6 shows images of the pelvic level, femoral level, and lower leg level of healthy subjects (volunteers). In contrast to the pathological condition, a clear symmetry, uniformity of the density of muscle tissue, organs of the pelvic level, and the absence of pathological formations draw attention.

## 4. Discussion

The study assessed the motor capabilities of patients with DMD who were capable or not capable of independent walking. In both groups, the greatest decrease in the ability to lift and move with the relative preservation of axial, proximal, and distal motor functions was revealed.

It is known that ultrasound assessment of skeletal muscle with advanced quantitative image analysis can be an effective alternative method for assessing skeletal muscle in DMD, especially in early childhood, when MRI of muscles without anesthesia is not possible. However, this method has limitations due to the difficulty in assessing the deep muscles of the thighs and pelvic girdle. In older patients with DMD, this method may have significant limitations due to the fact that being overweight is associated with corticosteroid therapy [33].

In the group of patients capable of independent walking, a decrease in the ability to lift and move (D1) was noted in tasks such as moving from a sitting position to a standing position, lifting from a chair, maintaining a standing position on one leg, tilting to the floor from a standing position, walking 10 steps on the heels, running a distance of 10 m, jumping on one leg, and squats. The decrease in axial and proximal motor functions (D2) was due to difficulties in performing tasks for lifting and tilting the head to the chest in the supine position, raising the legs in the supine position, and a coup from the back to the stomach. The decrease in the distal function indices (D3) was due to tasks involving extending the foot, as well as difficulties when trying to tear paper folded in four layers.

In the group of patients incapable of independent walking, the ability to lift and move was 1.7%. The safety in this subsection was determined by the task of moving from a lying position to a sitting position due to compensatory movements, resting on hands, or completing a task through a sideways flip. The relative preservation of axial and proximal motor functions (D2) was due to tasks involving turning the head to the side in the supine position, holding the position while sitting on a chair, raising the head in the sitting position, raising the arms to the head in the sitting position, and extending the elbow and lifting both hands at the same time on the table. The decrease in distal functions (D3) in this group was mainly due to the extension of the foot, the holding of 10 coins in the hand, the inability to tear paper folded in four or two layers, and the supination of the brush.

In our study, we performed a correlation analysis of the results of the MFM scale with the severity of fibro-fatty degeneration of the skeletal muscles of the pelvic girdle, thighs, and lower legs, estimated using the Mercuri scale, as well as with the age of the patients. A group of patients with intact mobility was characterized by a strong inverse correlation between the MFM scale and MRI results, which coincides with the data of Schmidt S., Hafner P., and others. The study showed a strong relationship between the results of quantitative MRI of skeletal muscles of the hips with the MFM scale (total and tasks D1) and a 6-min walk test. However, in this work, only children with the ability to move independently were examined at the age from 6.5 to 10.8 years (mean age—8.2 years) [34]. According to the data of research there was no correlation between the age of the patients and the data of the clinical evaluation; however, according to the results of our study, a high inverse correlation of these indicators was revealed [34]. The absence of a strong correlation in patients with young DMD may be due to a short period of improvement in motor skills and muscle strength [35,36]. The results of quantitative MRI study are characterized by a high positive correlation with age, which is also confirmed by a semi-quantitative assessment on the Mercuri scale in our study [34].

The data of our research coincide with the study, where a strong negative correlation of D1 indices and total MFM score (r = −0.9) with quantitative MRI of the thigh muscles was demonstrated [37]. However, in this work, a strong relationship is observed with tasks D2 (r = −0.8), while according to our data, only a noticeable correlation was noted in children who were able to move (r = −0.5) and a moderate correlation in patients incapable of independent movement (r = −0.4). This may be due to the lower sensitivity of semi-quantitative methods for assessing fatty infiltration, such as Mercuri and Lamminen scales, compared to quantitative MRI using the T2 mapping and 3-point Dixon protocols [38,39].

Quantitative MRI of the skeletal muscles of the thighs and legs in DMD is a proven effective biomarker of the severity of the disease, an effective method for dynamic follow-up. Quantitative MRI has a good correlation with motor tests such as the 6-min walk test (6MWT) and time tests (10 m run/walk, floor standing, stair climbing). One potential biomarker is vastus lateralis (VL) fat replacement. Additionally, quantitative MRI data showed a strong inverse correlation (r = −0.6, *p* < 0.05) with the NSAA scale. The results of our study also demonstrate a strong correlation of fibro-fatty degeneration of the muscles of the pelvic girdle, hips, and lower legs with motor abilities in patients at outpatient stages of the disease [40,41,42].

Thus, the use of semi-quantitative skeletal muscle imaging techniques can be an alternative method for assessing the severity of DMD at outpatient stages in conditions of technical limitations, when it is impossible to perform a quantitative assessment. However, according to the results of our study, there is no significant correlation in the non-ambulatory stages of DMD. This may be associated with severe motor impairments in the lower extremities in the later stages of the disease, and requires an additional study of the shoulder girdle and further comparison with the results of the assessment of subsections D1 and D2 of the MFM scale or other scales for assessing the motor abilities of the upper extremities, for example, Performance Upper Limb [10].

## 5. Conclusions

While maintaining the ability to move, the gmost information about a decrease in motor capabilities is provided by the task indicators, which are aimed at assessing verticalization and walking (D1) when assessing according to the MFM scale. For patients without the ability to move independently, the most informative were tasks aimed at assessing axial and proximal (D2), as well as distal (D3) motor capabilities.

The Motor Function Measure scale allows you to effectively assess the motor capabilities of patients with Duchenne muscular dystrophy at different stages of the disease, which is confirmed by visualization of fibro-fatty muscle replacement.

Correlation analysis in the study groups revealed the predominance of a high and noticeable strength of the relationship between quantitative MRI of the muscles of the pelvic girdle and hips with the MFM and MRC scales, as well as with the age of the patients (*p* = 0.01). Quantitative MRI of the lower leg muscles was characterized by a noticeable and moderate strength of correlation with these scales and a high correlation with the age of the patients (*p* = 0.01).

Semi-quantitative MRI of the muscles of the pelvic girdle, hips and lower legs in all studied groups was characterized by the predominance of a noticeable, moderate, and weak correlation with the MFM and MRC scales, and a high correlation with the age of patients.

Semi-quantitative skeletal muscle imaging techniques may be an alternative method for assessing the severity of DMD in outpatient stages in the context of technical limitations, when it is impossible to perform a quantitative assessment. However, our study did not show a significant correlation in the non-ambulatory stages of DMD. This may be associated with severe motor impairment in the lower extremities in the later stages of the disease, and requires additional study of the shoulder girdle and further comparison with the results of the assessment of subsections D1 and D2 of the MFM scale or other scales for assessing the motor abilities of the upper extremities, for example, Performance Upper Limb.

## Figures and Tables

**Table 1:** Mercuri Fibroid Fat Degeneration Scale.

Points	The Degree of Fibrous-Fatty Degeneration of Muscle Tissue
0	Normal muscle tissue
1	Initial manifestations of the “moth-eaten” phenomenon muscle fibers with small areas of increased MR signal
2a	Late manifestations of the phenomenon of “moth-eaten” muscle fibers with numerous separate areas of increase in the MR signal, starting to merge, involving up to 30% of the muscle volume
2b	Late manifestations of the phenomenon of “moth-eaten” muscle fibers with numerous separate areas of increase in the MR signal, starting to merge, involving 30–60% of the muscle volume
3	The appearance of blurriness and fuzziness due to the fusion of at least areas in one muscle with an increase in the MR signal
4	The final stage of muscle tissue degeneration—the replacement of its connective and adipose tissues with an increased MR signal—while the fascia rings and neurovascular bundles are distinguishable

**Table 2:** General quantitative characteristics of the study.

	Ambulatory DMD Patients	Non-Ambulatory DMD Patients
Participants (*n* = )	34	7
Age (mean) (y)	6.7	13.9
Age minimum/maximum (y)	2.1/11.5	10.2/16.7
Steroid treatment (*n* = /%)	28/82.4%	3/42.9%
Sex: male/female (*n* =)	34/0	7/0
Ethnicity: Caucasian (%)	100%	100%
Motor functions:		
Ability to stand up without Gowers signs (*n* = /%)	4/11.8%	0/0%
Ability to stand up with Gowers signs (*n* = /%)	30/88.2%	0/0%
Ability to run 10 m without compensatory movements (*n* = /%)	11/32.4%	0/0%
Ability to run 10 m with compensatory movements/walk (*n* = /%)	23/67.7%	0/0%
Ability to sit from supine position (*n* = /%)	34/100%	2/28.6%

**Table 3:** Correlation analysis (r_Spearman_) of the MFM scale and the results of MRI of the muscles of the pelvic girdle, hips, and lower legs (Mercuri scale assessment of fibro-fat degeneration) in patients who are able to move independently.

	Pelvic Muscles MRI	Femoral Muscles MRI	Calf Muscles MRI	MRI Scores, Total
MFM D1 (lifting and moving)	r = −0.7 *p* ≤ 0.01	r = −0.7 *p* ≤ 0.01	r = −0.6 *p* ≤ 0.05	r = −0.7 *p* ≤ 0.01
MFM D2 (axial and proximal motor functions)	r = −0.5 *p* ≤ 0.05	r = −0.5 *p* ≤ 0.05	r = −0.3	r = −0.5 *p* ≤ 0.05
MFM D3 (distal motor function)	r = −0.3	r = −0.2	r = −0.6 *p* ≤ 0.05	r = −0.6 *p* ≤ 0.05
MFM, total score	r = −0.6 *p* ≤ 0.05	r = −0.7 *p* ≤ 0.01	r = −0.5 *p* ≤ 0.05	r = −0.6 *p* ≤ 0.05

**Table 4:** Correlation analysis (r_Spearman_) of the MFM scale and the results of MRI of the muscles of the pelvic girdle, thighs, and lower legs (Mercuri scale assessment of fibro-fat degeneration) in patients incapable of independent movement.

	Pelvic Muscles MRI	Femoral Muscles MRI	Calf Muscles MRI	MRI Scores, Total
MFM D1 (lifting and moving)	r = −0.3	r = −0.3	r = −0.6 *p* ≤ 0.05	r = −0.3
MFM D2 (axial and proximal motor functions)	r = −0.4	r = −0.4	r = −0.2	r = −0.3
MFM D3 (distal motor function)	r = −0.2	r = −0.2	r = −0.7 *p* ≤ 0.05	r = −0.3
MFM, total score	r = −0.3	r = −0.2	r = −0.3	r = −0.5 *p* ≤ 0.05

**Table 5:** Correlation analysis (r_Spearman_) of the age of patients capable and not capable of independent movement with the results of the assessment according to the MFM scale and MRI data.

	Age of Patients with Intact Mobility	Age of Patients without the Ability to Move Independently
MFM D1 (lifting and moving)	r = −0.7, *p* ≤ 0.01	r = −0.7, *p* ≤ 0.01
MFM D2 (axial and proximal motor functions)	r = −0.7, *p* ≤ 0.01	r = −0.8, *p* ≤ 0.01
MFM D3 (distal motor function)	r = −0.5, *p* ≤ 0.05	r = −0.9, *p* ≤ 0.01
MFM, total score	r = −0.7, *p* ≤ 0.01	r = −0.9, *p* ≤ 0.01
Pelvic muscles MRI	r = 0.7, *p* ≤ 0.01	r = 0.7, *p* ≤ 0.01
Femoral muscles MRI	r = 0.7, *p* ≤ 0.01	r = 0.7, *p* ≤ 0.01
Calf muscles MRI	r = 0.5, *p* ≤ 0.05	r = 0.9, *p* ≤ 0.01
MRI scores, total	r = 0.7, *p* ≤ 0.01	r = 0.8, *p* ≤ 0.01

**Table 6:** Evaluation of the severity of fibro-fatty muscle degeneration according to the Mercuri scale (point) of patients in accordance with clinical examples Figure 3, Figure 4 and Figure 5.

Muscle Groups	Patient, 6.1 Years (Figure 3)		Patient, 9.4 Years (Figure 4)		Patient, 13 Years (Figure 5)	
	Left	Right	Left	Right	Left	Right
m.gluteus maximus	2.5	2.5	3	3	4	4
m.gluteus medius	2.0	2.0	2.5	2.5	4	4
m.gluteus minimus	2.0	2.0	2.0	2.0	3	3
m.adductor longus	0	0	1	1	3	3
m.adductor brevis	0	0	0	0	3	3
m.sartorius	1	1	1	1	4	4
m.gracilis	0	0	1	0	2.0	1
m.adductor magnus	2.5	2.5	3	3	4	4
m.vastus lateralis	2.0	1	2.0	2.0	4	4
m.vastus medialis	2.0	2.0	2.0	2.0	4	4
m.vastus intermedius	2.0	2.0	2.0	2.0	4	4
m.rectus femoris	1	1	2.5	2.5	4	4
m. semitendinosus	2.0	2.0	2.0	2.0	4	4
m. semimembranosus	1	1	2.0	2.0	4	4
m. biceps femoris	2.0	1	2.5	2.5	4	4
m. gastrocnemius	1	1	2.0	2.0	3	3
m. soleus	2.0	2.0	2.0	2.0	4	4
m. peroneus	1	1	3	3	4	4
m. tibialis anterior	1	1	1	1	2.5	2.0
m. tibialis posterior	0	0	0	0	0	0
m. extensor digitorum longus	0	0	0	0	1	1
m. extensor hallucis longus	0	0	0	0	1	1
m. flexor digitorum longus	0	0	0	0	2.5	2.5
m. flexor hallucis longus	0	0	0	0	2.5	2.5

## Data Availability

Not applicable.
